# Informing Selection of Nanomaterial Concentrations for ToxCast *in Vitro* Testing Based on Occupational Exposure Potential

**DOI:** 10.1289/ehp.1103750

**Published:** 2011-07-25

**Authors:** Sumit Gangwal, James S. Brown, Amy Wang, Keith A. Houck, David J. Dix, Robert J. Kavlock, Elaine A. Cohen Hubal

**Affiliations:** 1 National Center for Computational Toxicology, and; 2 National Center for Environmental Assessment, Office of Research and Development, U.S. Environmental Protection Agency, Research Triangle Park, North Carolina, USA

**Keywords:** ExpoCast, human particle deposition and retention, *in vitro* nanomaterial concentration, multiple-path particle dosimetry (MPPD), occupational exposure, ToxCast

## Abstract

Background: Little justification is generally provided for selection of *in vitro* assay testing concentrations for engineered nanomaterials (ENMs). Selection of concentration levels for hazard evaluation based on real-world exposure scenarios is desirable.

Objectives: Our goal was to use estimates of lung deposition after occupational exposure to nanomaterials to recommend *in vitro* testing concentrations for the U.S. Environmental Protection Agency’s ToxCast™ program. Here, we provide testing concentrations for carbon nanotubes (CNTs) and titanium dioxide (TiO_2_) and silver (Ag) nanoparticles (NPs).

Methods: We reviewed published ENM concentrations measured in air in manufacturing and R&D (research and development) laboratories to identify input levels for estimating ENM mass retained in the human lung using the multiple-path particle dosimetry (MPPD) model. Model input parameters were individually varied to estimate alveolar mass retained for different particle sizes (5–1,000 nm), aerosol concentrations (0.1 and 1 mg/m^3^), aspect ratios (2, 4, 10, and 167), and exposure durations (24 hr and a working lifetime). The calculated lung surface concentrations were then converted to *in vitro* solution concentrations.

Results: Modeled alveolar mass retained after 24 hr is most affected by activity level and aerosol concentration. Alveolar retention for Ag and TiO_2_ NPs and CNTs for a working-lifetime (45 years) exposure duration is similar to high-end concentrations (~ 30–400 μg/mL) typical of *in vitro* testing reported in the literature.

Conclusions: Analyses performed are generally applicable for providing ENM testing concentrations for *in vitro* hazard screening studies, although further research is needed to improve the approach. Understanding the relationship between potential real-world exposures and *in vitro* test concentrations will facilitate interpretation of toxicological results.

Researchers evaluating toxicity and human exposure potential of engineered nanomaterials (ENMs) are challenged by rapid development of novel materials for new applications as the nanotechnology industry drives forward. These materials can add significant value to industrial or consumer products. ENMs have one or more components with at least one dimension in the range of 1–1,000 nm. Components can include nanoparticles (NPs), nanofibers and nanotubes, nanodots, nanostructured surfaces, or nanocomposites. Carbon nanotubes (CNTs) and metal oxide NPs (two material types having the highest industrial production volumes) are used in plastics, catalysts, battery and fuel cell electrodes, solar cells, paints, coatings, etc. (Klaine et al. 2008). Nanoparticulate silver (Ag) has the greatest number of consumer product applications. Novel nanomaterial (NM) types continue to be synthesized based on the value they may add, often without evaluation of implications for human health, toxicity, environmental impact, or long-term sustainability. NMs, especially the ones made of metals, semiconductors, and various inorganic compounds, have the potential for postuse risks to humans and the environment (National Nanotechnology Initiative 2008). These concerns need to be examined and addressed before the widespread adoption of nanotechnologies (Oberdörster et al. 2005).

The U.S. Environmental Protection Agency (U.S. EPA) is beginning to evaluate exposure and hazard potential of NMs and prioritize them for further animal-based toxicological testing. Prioritization of NM classes and types for targeted testing is important in the early stages of NM development. Currently, only a small portion of the thousands of commonly used chemicals in the Toxic Substances Control Act (1976) inventory (U.S. EPA 2004) have undergone animal testing because of the high cost (millions of dollars) and long time frame (2–3 years) required per chemical (Judson et al. 2009). Of the unique chemicals (~ 10,000) the U.S. EPA is most concerned with, only a fraction have been evaluated for specific classes of toxicity (Judson et al. 2009). The ToxCast research program of the U.S. EPA was started in 2007 and seeks to predict the potential toxicity of environmental chemicals based on *in vitro* bioactivity profiling at minimal cost compared with full-scale animal testing (Dix et al. 2007). An initial set of approximately 300 chemicals (primarily pesticides) was tested in phase I of ToxCast in 467 high-throughput screening (HTS) biochemical and cell-based assays across nine technologies (Judson et al. 2010). A study has been initiated to evaluate the potential of ToxCast methods for screening NMs. A subset of ToxCast *in vitro* HTS cell-based assays will be run on NMs to produce similar bioactivity profiles and toxicity predictions. Most of the cell-based assays have an exposure time of 24 hr. Initial NM types to be evaluated include single-walled carbon nanotubes (SWCNTs) and multiwalled carbon nanotubes (MWCNTs), along with Ag, titanium dioxide (TiO_2_), and gold (Au) NPs.

Design and conduct of ToxCast screening of NMs requires selection of testing concentrations, characterization of materials, and analysis of resulting HTS data. Selection of concentrations used for *in vitro* toxicity studies of NMs often lacks scientific justification, and concentrations are often chosen to be very high to ascertain a toxicological end point without consideration of real-world exposure (Oberdörster et al. 2005). Some researchers have used particle concentrations causing “overload” (Warheit et al. 2009), a dose where pulmonary clearance becomes severely impaired (Morrow 1988). Although high testing concentrations may be considered to ensure that NMs show bioactivity across the spectrum of assays evaluated, there is also a need for biologically relevant human exposure information to facilitate interpretation of assay results (Cohen Hubal 2009). Authors of *Toxicity Testing in the 21st Century: A Vision and a Strategy* (National Research Council 2007) noted that human exposure information is required to select doses for toxicity testing, facilitating development of environmentally relevant hazard information.

Recognizing the critical need for exposure information to inform chemical design, evaluation, and health risk management, the U.S. EPA ExpoCast^TM^ program was initiated in 2010 to meet challenges posed by new toxicity testing approaches (Cohen Hubal et al. 2010). The goal of ExpoCast is to advance characterization of exposure required to translate findings in computational toxicology to information that can be directly used to support exposure and risk assessment. Combining information from ToxCast with information from ExpoCast will help the U.S. EPA prioritize NMs and chemicals for further evaluation based on potential risk to human health.

Human exposures to ENMs are likely to be higher for workers in occupational settings than for the general population, including consumers (Bergamaschi 2009), and may thus provide upper bounding estimates of exposure potentials. For consumers, the greatest exposure to ENMs likely comes from products that are ingested or that come into intimate contact with the body (Kessler 2011). Although ingestion and dermal exposures must also be considered during the product life cycle (manufacturing, usage, and disposal of EMNs) (Oberdörster et al. 2005; U.S. EPA 2010), inhalation may be the key route of human exposure in nanotechnology manufacturing and R&D (research and development) facilities (Bergamaschi 2009; Hoet et al. 2004). Many studies have focused on the inhalation exposure route for ENMs and have considered potential airborne releases of NMs from facilities. After intake of NP-containing aerosols, high deposition fractions in the alveolar region (for particles < 100 nm in size) and the head region (diameter < 5 nm) are predicted by the multiple-path particle dosimetry (MPPD) and International Commission on Radiological Protection (ICRP) models (U.S. EPA 2009). NM exposure of the lung parenchyma is of concern because of long-term retention in this lower region and potential for particles to cause cytotoxicity and translocate.

## General Approach

**Figure 1:**
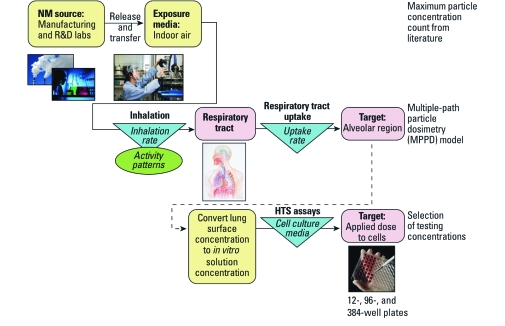
General approach for recommending *in vitro* testing levels, considering exposure to NMs from occupational-setting indoor air via the inhalation route resulting in respiratory tract uptake. Estimated exposure potential is converted to levels for NM testing in HTS cellular assays.

The aim of this study was to use information on potential ENM exposure in the occupational setting to recommend *in vitro* testing levels for bioprofiling in U.S. EPA ToxCast program. Our general approach (Figure 1) was to assume that the inhalation exposure route for NMs is of primary concern for humans in occupational settings. Occupational aerosol levels of NMs reported in the literature were reviewed and used as inputs for lung dosimetry modeling. We assumed that these reported NM concentrations from manufacturing and R&D laboratory facilities would provide a high-end potential for real-world NM exposure to the general population, higher than exposures that may result from consumer products (Bergamaschi 2009).

We used the maximum reported NM aerosol concentrations (mass per cubic meter of air) as an input for the MPPD model to estimate deposition, clearance, and mass retained in the alveolar region of the human lung. A sensitivity analysis was performed to evaluate MPPD input parameters that most affect NM alveolar retention after 24 hr of exposure. Two exposure scenarios were considered for further modeling: exposure over the course of 24 hr (based on the standard assay exposure duration) and 45 years (a full occupational lifetime). For each scenario, we varied the significant parameters to estimate the mass of particles retained in the alveolar region per surface area. Model results of lung surface mass concentrations were then converted (using the reported well-bottom surface area and volume delivered) to suggest testing solution mass concentrations for *in vitro* screening. All of the applied material was assumed to deposit on the bottom of the well. The results suggest upper and lower bounding HTS assay testing concentrations based on potential for real-world NM exposures at short and long durations via the inhalation route in an occupational setting. The concentrations were subsequently compared with *in vitro* concentrations found in recent literature. Although we have chosen here to consider aerosol mass concentration, we recognize that other lung deposition metrics (based on particle number or particle surface area) are also potentially important for understanding health risk.

A small fraction of NPs deposited in the alveolar region may be cleared into the bloodstream by absorption. Particles that deposit in the respiratory tract can also be cleared to the gastrointestinal (GI) tract via the pharynx or to the regional lymph nodes (LN) via lymphatic channels. Only lung surface cells would receive the same concentration of NPs as estimated here for inhalation. Modeling exposure to other cell types is beyond the scope of this article, but the concentrations from these exposures would likely be significantly lower than those calculated for lung cells.

## Data and Methods

*NM air concentrations.* We reviewed occupational exposure studies that measured airborne levels of ENMs. The instruments used to obtain particle number concentrations were typically the condensation particle counter (CPC), scanning mobility particle sizer (SMPS), and the fast mobility particle sizer (FMPS). In some cases, personal air samplers collected NPs on filters from the breathing zone of workers during the work day. The SMPS and FMPS instruments provide real-time temporal changes in particle size. The data give particle number concentrations (particles per cubic centimeter of air) versus particle diameter across the size distribution. The CPC provides particle number concentration (particles per cubic centimeter of air) for particles in the range of 2.5 to > 1,000 nm. The instruments can also report the change in particle number concentration versus time. We searched for the highest aerosol particle number concentrations for TiO_2_ and Ag NPs and for CNTs (including MWCNTs) in manufacturing and R&D settings (Table 1). Background particle number concentrations were subtracted from the maximum particle number concentrations if they were reported. Typically, particle counts per volume (cubic centimeters) of air are reported, whereas exposure limits are set as mass concentrations (milligrams per cubic meter). To convert from reported particle count concentration to mass concentration, TiO_2_ and Ag NPs were assumed to be spherical, and the reported size (taken to be geometric particle diameter) was used to calculate a particle volume. The CNTs were assumed to be cylindrical, and reported diameter and length were used to obtain particle volume. We assumed a CNT length of 0.5 μm if it was not reported. A density of 4, 10, and 2 g/cm^3^ was assumed for TiO_2_ NPs, Ag NPs, and CNT, respectively, based on specifications of similar materials from supplier web sites (Nanostructured & Amorphous Materials Inc. 2010; Sigma-Aldrich 2010). The high-end reported particle counts were approximated to mass concentrations by multiplying particle volume by the assumed density (Table 1).

**Table 1:** NM exposure concentrations in lab and manufacturing sites.

NM	Highest particle count (no./cm^3^)	Mnfg/lab	Particle size (nm)	Calculated mass concentration (mg/m^3^)	Instrument used for detection	Reference
Ag NPs		72,900		Mnfg		35		0.02		CPC		Methner et al. 2010a, 2010b
		1.102 × 10^7^ (for 55 min)		Mnfg		76		0.46 (for 1 min)		SMPS		Park et al. 2009
		7,000		Lab		150		0.12		FMPS		Tsai et al. 2009
		995,000 (for 15–30 min)		Mnfg		10		0.005		FMPS		Miller et al. 2010
								0.094 (for full work shift)		Personal filter sample		
								[0.01 (OSHA PEL; airborne Ag)]				
MWCNTs		35,800		R&D Lab		20 nm diameter, 0.5 μm length		0.01		CPC		Methner et al. 2010a, 2010b
		—		—		—		[0.05 (researcher-suggested OEL)]		—		Pauluhn 2010
		75,000; during metal catalyst preparation		Mnfg – seven sites		25 nm diameter, no reported length		0.037		SMPS		Lee et al. 2010
								0.32		Personal air		
CNTs		7,000; no detectable CNTs or bundles		Lab		10 and 100 diameter		0		FMPS and CPC		Bello et al. 2008
		5,000,000 (CNT carbon composites; no detectable CNTs or bundles)		R&D Lab		—		0		FMPS		Bello et al. 2009

		—		—		—		0.007 (draft REL for elemental carbon)		—		NIOSH 2010
TiO_2_ NPs		111,300		Mnfg		40		0.015		CPC		Methner et al. 2010a, 2010b
		—		—		—		0.1 (draft REL)		—		NIOSH 2005
		140,000		Mnfg		16		0.001		SMPS		Hameri et al. 2009
Abbreviations: Mnfg, manufacturing; OEL, occupational exposure limit.												

The calculated mass concentrations were typically less than approximately 0.1 mg NM per volume (cubic meters) of air (Table 1). One study on MWCNTs reported a higher mass concentration of 0.3208 mg/m^3^ (Lee et al. 2010). However, this value was from personal sampler filters with typical sampling durations of 183–409 min. The mass concentration would be lower if calculated over the time duration. Data normalized over exposure characterization duration from a liquid-phase production facility of Ag NPs yielded a mass concentration of 0.46 mg/m^3^ for 1 min (Park et al. 2009). In that study, both change in particle number concentration versus time and total number of particles (with diameters between 10 and 250 nm) counted over a range of time were reported. A conservative aerosol concentration of 1 mg/m^3^ was taken to be an upper exposure limit. Although the U.S. National Institute for Occupational Safety and Health (NIOSH) does not have a recommended exposure limit (REL) for TiO_2_ NPs, a draft NIOSH bulletin (NIOSH 2005) recommended “0.1 mg/m^3^ for ultrafine TiO_2_, as time-weighted average concentrations (TWA) for up to 10 hr/day during a 40-hour work week,” where “ultrafine” is defined as the fraction of respirable particles with primary particle diameter < 100 nm. A recent draft NIOSH bulletin (NIOSH 2010) proposed a REL of 0.007 mg/m^3^ for CNTs and carbon nanofibers. Using a different approach, an occupational exposure limit of 0.05 mg/m^3^ was derived for Baytubes, a more flexible MWCNT type (Pauluhn 2010). There is no limit set for Ag NPs in the United States. However, the Occupational Safety and Health Administration (OSHA) established a permissible exposure limit (PEL) of 0.01 mg/m^3^ (which is the same as the REL set by NIOSH) for all forms of airborne Ag (Miller et al. 2010). The American Conference of Governmental Industrial Hygienists (ACGIH) set a threshold limit value (TLV) of 0.1 mg/m^3^ for metallic Ag and 0.01 mg/m^3^ for soluble Ag compounds (Miller et al. 2010). In the present study, the mass concentrations derived based on measured aerosol levels were taken as a basis and used as inputs to model the mass of NPs that could deposit and be retained deep in human lungs.

*Lung dosimetry modeling.* MPPD model application. We estimated particle deposition and clearance in human lungs using the recently developed, publicly available MPPD model (version 2.1 for NPs, presently supported by Applied Research Associates Inc., Raleigh, NC). The model can be used to estimate particle dosimetry in both human and rat airways (Anjilvel and Asgharian 1995; Asgharian et al. 2001). It calculates deposition and clearance of particles ranging from ultrafine (0.001 μm) to coarse (100 μm) in the respiratory tract, based on user-provided input on airway morphometry, clearance rates, particle properties (density, diameter, and size distribution), and exposure scenario (aerosol concentration, activity breathing pattern, and exposure duration). Three main particle deposition mechanisms (impaction, sedimentation, and diffusion) are incorporated in the model, and deposition in different regions of the lung are calculated using published analytic formulas (Anjilvel and Asgharian 1995). Clearance from each lung region is treated competitively between absorption into the blood and particle transport processes (from the respiratory tract to the GI tract and to lymph nodes, and from one region to another) (ICRP 1994). Retention in the human alveolar-interstitial region is represented by three compartments, which clear at fast, medium, and slow rates to the lymph nodes and the bronchiolar region (ICRP 1994). Although the clearance kinetics in the MPPD model were based on studies of microsized particles, evidence suggests efficient surface macrophage uptake and clearance of both microparticles and NPs as well as penetration of both sizes of particles through the human lung epithelium into the interstitial region, from which they are slowly cleared (Geiser and Kreyling 2010). In addition, the MPPD model (version 2.1) incorporates improved estimates of particle losses from the airway by diffusion and includes particle-specific axial diffusion and dispersion effects in the transport equation (Asgharian and Price 2007). This updated model provides for more realistic assessment of regional deposition of diffusion-dominated (nanosized) particles in the lung (Asgharian and Price 2007). A MPPD model version (obtained directly from Applied Research Associates, Inc.) that incorporates length-to-diameter aspect ratio to predict inhaled nanofiber/nanotube deposition in the human lung was used for nonspherical CNTs/MWCNTs (NIOSH 2008). This model incorporates altered analytical expressions for deposition efficiency of nanofibers of a given aspect ratio by adjusting for the viscous drag and nanofiber orientation in the deposition efficiency equation for spherical particles. The clearance calculations are valid only for spherical particles.

We selected an initial baseline set of MPPD inputs (Table 2) based on data from the ICRP report (ICRP 1994), which provided morphological characteristics and physiological parameters for the human respiratory tract. We organized the MPPD model input parameters into three categories: individual characteristics, exposure scenario, and material properties. For the individual characteristics input, the airway morphometry selected was the human Yeh/Schum symmetric lung model (Yeh and Schum 1980). Default values were selected for the clearance rates and other parameters. For the exposure scenario input, 0.1 mg/m^3^ aerosol concentration was selected, and light exercise activity breathing pattern for an adult male was assumed with 20 breaths/min frequency at 1,250 mL tidal volume, (V_T_) (ICRP 1994). Oronasal-mouth breather was selected for breathing scenario, because humans typically switch to breathing partly through the mouth and through the nose at ventilation rates between light and heavy exercise (ICRP 1994). For the particle properties input, we selected a particle count median diameter of 40 nm, assuming a single mode of log-normal size distribution with size geometric standard deviation (GSD) of 1.25 based on the ICRP report. Inhalability was not considered because it approaches 100% for small (< 5 μm) particles (ICRP 1994). The length-to-diameter aspect ratio was set to 1.

**Table 2:** MPPD baseline settings.

MPPD baseline input categories	Baseline input settings
Individual characteristics (airway morphometry and deposition/clearance)		Human species; Yeh-Schum symmetric single path lung model; FRC = 3,300 mL; URT volume = 50 mL Tracheal mucous velocity = 5.5 mm/min; fast human clearance rate = 0.02/day; medium human clearance rate = 0.001/day; slow human clearance rate = 0.0001/day; lymph node human clearance rate = 0.00002/day
Exposure scenario: constant exposure		Acceleration of gravity = 981.0 cm/sec^2^; body orientation = upright; aerosol concentration = 0.1 mg/m^3^; breathing frequency = 20/min; V_T_ = 1,250 mL; inspiratory fraction = 0.5; pause fraction = 0; breathing scenario, oronasal-mouth breather; number of hours per day = 24; number of days per week = 1; number of weeks = 1; maximum postexposure days = 0
Particle properties		Density = 4 g/cm^3^; diameter = 0.04 μm; count median diameter checked; NP model checked; inhalability adjustment not checked; GSD (diameter) = 1.25
Abbreviations: FRC, functional residual capacity; URT, upper respiratory tract.		

Sensitivity analysis. Key determinants of MPPD model predictions of mass (milligrams) retained in the alveolar region were determined by systematically altering each input baseline parameter one at a time, while holding the others constant, and rerunning the model based on a 24-hr exposure duration with 1 week of total time (Table 3) to allow for clearance. For the individual characteristic inputs, we evaluated two different size (based on total number of airways) human stochastic lung models because they provide more realistic lung geometry than the symmetric lung model. Calculations were also performed using an age-specific symmetric lung model for a 3-year-old child. Although this group is unlikely to be exposed occupationally, we wanted to check model results for a vulnerable population group. The alveolar interstitial rate constants for fast, medium, slow, and lymph node human clearance were doubled, halved, increased by an order of magnitude, and decreased by an order of magnitude. Tracheal mucosal velocity was not considered because it affects only tracheobronchial clearance rates and residence times and will not affect long-term alveolar burden. For the exposure scenario inputs, the aerosol concentration was decreased by one order of magnitude from 0.1 mg/m^3^. As a conservative estimate in case the mass per air volume concentration was much higher than reported, the aerosol mass concentration was also increased by one and two orders of magnitude. Both heavy-exercise and resting breathing patterns were evaluated, as well as purely nasal and oral breathing. For the particle properties inputs, we considered a low size diameter of 5 nm, a high diameter of 100 nm, a low GSD of 1 (monodisperse diameter distribution), and a high GSD of 4 (polydisperse diameter distribution). Additionally, aspect ratios from 4 to 1,000 were evaluated with a length GSD of 1.0 (as a conservative estimate) and a density of 2.

**Table 3:** MPPD sensitivity analysis.

MPPD input category	Altered input setting	Output alveolar mass retained (mg)	Percent change in output	Percent change in input	Sensitivity percent
Individual characteristics		Human, stochastic lung model								
		1st size percentile		0.81		–34.1		—		—
		60th size percentile		1.01		–17.2		—		—
		Age-specific symmetric model:								
		3-year-old child		0.16		–87.1		—		—
		Clearance rates								
		0.1 × default alveolar-interstitial rate constants		1.27		3.8		–90		–4.18
		0.5 × default rate constants		1.25		2.0		–50		–4.09
		1.5 × default rate constants		1.20		–2.0		50		–3.92
		2 × default rate constants		1.18		–3.8		100		–3.76
		10 × default rate constants		0.95		–23.0		900		–2.50
Exposure scenario		Aerosol concentration								
		0.01 mg/m^3^		0.12		–90.0		–90		100
		1 mg/m^3^		12.2		898		900		99.7
		10 mg/m^3^		122.3		9,900		9,900		100
		Breathing pattern								
		Resting, 12 breaths/min; 625 mL V_T_		0.32		–74.1		–70		106
		Heavy exercise, 26 breaths/min; 1,923 mL V_T_		2.30		88.0		100		88.0
		Breathing scenario								
		Nasal		1.20		–2.1		—		—
		Oral		1.25		2.0		—		—
Particle properties		Diameter								
		5 nm		0.81		–33.9		–87.5		38.7
		20 nm		1.51		23.5		–50		–46.9
		50 nm		1.07		–12.7		25		–50.7
		70 nm		0.85		–30.7		75		–41.0
		100 nm		0.65		–46.6		150		–31.1
		GSD								
		1		1.33		9.08		–20		–45.4
		1.6		0.90		–26.7		28.0		–95.5
		2		0.60		–51.2		60		–85.3
		2.8		0.68		–44.3		124		–35.7
		4		0.12		–90.1		220		–41.0
		Aspect ratio (length:diameter)								
		1 (Baseline) (20 nm:20 nm)		1.54		0		0		—
		4 (80 nm:20 nm)		1.26		–18.3		300		–6.09
		20 (400 nm:20 nm)		1.56		1.69		1,900		0.09
		100 (2 μm:20 nm)		1.12		–27.3		9,900		–0.28
		500 (10 μm:20 nm)		0.61		–60.1		49,900		–0.12
		1,000 (20 μm:20 nm)		0.54		–65.0		99,900		–0.07
—, not applicable.										

For this sensitivity analysis, if the alveolar mass retained using the new setting resulted in a percentage change ≥ 10% of the baseline amount, the parameter was considered to be significant and was evaluated further. If the alveolar mass retained using the new setting yielded a negative percentage change compared with the baseline setting, then the input was not considered, because we are interested in a conservative exposure approach that may overestimate particle deposition and retention deep in the lungs. If alveolar retention output did not change linearly with change in input, additional input changes were considered to better characterize model behavior over the relevant range. The MPPD input parameters determined to be significant were evaluated further to calculate mass retained in the alveolar region per alveolar surface area, based on two exposure durations: a short-term exposure duration of 24 hr and a long-term occupational lifetime exposure. The long-term scenario assumed a 45-year full working lifetime (Schulte et al. 2010) with 8 hr inhalation per day, 5 days/week, 52 weeks/year. The alveolar surface area (~ 106,350 cm^2^) was obtained from the MPPD model results report by summing the pulmonary surface area for lung generations 17 to 24. This alveolar surface area accounts for only surface area of the airways (alveolar ducts) and not the alveolar sacs, and thus is a low estimate of the actual alveolar surface area. The MPPD calculations were performed for different particle sizes (5, 10, 20, 30, 40, 50, 60, 70, and 100 nm), aerosol concentrations (0.1 and 1 mg/m^3^), and exposure durations. Larger particle sizes (200, 500, and 1,000 nm) were also run because particle aggregation of nanosized particles may occur in air (Maynard et al. 2004; Methner et al. 2010a) or inside the human respiratory tract. For CNTs, an aspect ratio of 167 was selected based on material dimensions (5 μm length, 30 nm diameter) of one sample to be tested in ToxCast. Aspect ratios of 2, 4, and 10 were also run for the different particle sizes. These aspect ratios were chosen based on electron images of SWCNT aggregates from the literature (Baron et al. 2008). Searching for realistic airborne CNT aspect ratios was challenging because many exposure studies found no evidence of carbon-based nanotubes or nanotube bundles in air samples (Bello et al. 2008, 2009). In one study of seven CNT-handling workplaces, transmission electron micrographs reveal clumped structures with aspect ratios of approximately 8–10 and diameters of approximately 100 nm (Lee et al. 2010). However, these particle aggregates are mostly metal components rather than CNTs.

*Determining* in vitro *concentrations.* Based on MPPD model predictions, we determined associated *in vitro* concentrations by calculating mass retained in the alveolar region of the lung per alveolar surface area for each particle size, at two aerosol concentrations (0.1 and 1 mg/m^3^) and for each exposure scenario (24 hr and 45 years). We assumed that the NM mass retained at the lung surface can be directly correlated to NM mass sedimented on the bottom surface of a well. To convert to mass of NM per volume of solution, we multiplied the resulting mass per alveolar surface area concentration (micrograms per square centimeter) by the bottom surface area of a single well in a 12-, 96- or 384-well plate and divided by the volume of culture medium added to each well (as obtained from the assay contractors). The 12-, 96-, and 384-well plates had a single well-bottom surface area of 3.8, 0.32, and 0.056 cm^2^ and volume of 1,000, 200, and 50 μL, respectively. The converted concentrations were compared with *in vitro* concentrations tested using human, mouse, and rat cell lines in the literature [see Supplemental Material, Tables S1–S3 (http://dx.doi.org/10.1289/ehp.1103750)]. Although final selection of concentration will include consideration of the MPPD model output and conversion, there will still be a need to test at levels based on where bioactivity has been demonstrated in the literature, bounded by concentration levels that can be dispersed with long-term stability in cell culture media.

Another method to determine high range *in vitro* concentrations to test could be to evaluate a NM steady-state mass in the alveolar region of the lung. Steady state occurs when the clearance rate equals the rate of deposition and the NM mass retained reaches a constant value. According to Brown et al. (2005), it takes > 10 years to reach a steady-state lung burden for insoluble 1 μm-sized particles for a 0.01-mg/m^3^ aerosol concentration based on resting human breathing pattern.

## Results and Discussion

*Key MPPD model input parameters.* For the MPPD baseline settings used here (Table 2), a steady-state retention dose would take > 80 years to achieve for 40-nm particles based on inhalation of an aerosol concentration of 0.1 mg/ m^3^ for 8 hr/day, 5 days/week. Because of the long time to achieve steady state, we did not use that method. Instead, we focused on modeling potential exposure scenarios and understanding implications of associated model inputs. Model results for the baseline input parameters (Table 2) resulted in 1.22 mg alveolar mass retained.

Results of the sensitivity analysis [alveolar mass retained, percentage change in model output and input, and sensitivity percentage (output percentage change by input percentage change)] are presented in Table 3. Although interactions between input parameters may occur, we assumed that key parameters could be uncovered by varying one parameter per run. Based on this analysis, aerosol concentration and heavy-exercise breathing pattern were the most important MPPD input parameters, as these increased alveolar retention by > 10%. For variations to the inputs for individual characteristics (Table 3), the choice of airway morphometry using the the human stochastic lung model resulted in a lower retention compared with the symmetric lung model. The age-specific symmetric lung model for a 3-year-old child resulted in lower mass retained at 0.16 mg because of lower intake (functional residual capacity, upper respiratory tract volume, and V_T_) compared with the adult male default baseline condition. Thus, the Yeh/Schum symmetric model provided a conservative estimate of NM particle dosimetry. Nasal and oral breathing scenario did not significantly affect the results and was set to the baseline of oronasal breathing. Increasing and decreasing the default alveolar-interstitial rate constants did not significantly affect the result as indicated by the sensitivity percentage in Table 3. The alveolar-interstitial rate constants were set to the default values and the lung model to symmetric to further calculate alveolar mass retained per alveolar surface area.

For the exposure scenario inputs (Table 3), the correlation between alveolar mass retained and aerosol concentration was linear: The amount retained in the alveolar region changed linearly by one order of magnitude as the exposure aerosol concentration (and thereby the intake) was increased or decreased by one order of magnitude, yielding a sensitivity of 100%. Using resting or heavy-exercise breathing pattern resulted in a sensitivity of approximately 100%, indicating that alveolar mass retained changed almost linearly with minute ventilation (breathing frequency by V_T_). To further calculate alveolar mass retained per alveolar surface area, the breathing scenario was set to light exercise, based on the assumption that this was the most realistic for a full working lifetime. The aerosol concentration of 1 mg/m^3^ was taken as a conservative estimate of potential worker exposure.

Particle property input changes to particle diameter, size GSD, and aspect ratio (length:diameter) did not result in linear changes to output alveolar mass retention, as observed in the sensitivity percent column in Table 3. Particle diameter of 20 nm resulted in maximum alveolar mass retained of 1.51 mg for diameters between 5 and 100 nm and was approximately 24% increase in output compared with baseline 40 nm size. A GSD value of 1 (monodisperse size distribution) yielded a higher mass amount retained, but it was only 9.08% more than the baseline-size GSD value of 1.25 and did not meet the sensitivity analysis requirements. All other input changes for diameter and GSD lowered the alveolar mass retained compared with the baseline settings. In a report on TiO_2_ particles, Hameri et al. (2009) listed a GSD of 1.66, and in a study of Ag NPs, Park et al. (2009) listed GSD values of 4.63–6.3. Although a higher-size GSD value is expected for realistic size distribution of NMs, this parameter was set to 1.25 as a conservative estimate that would increase alveolar retention. Changes to the aspect ratio input at constant aerosol concentration and minute ventilation lowered the alveolar mass retained compared with the baseline (aspect ratio 1 in Table 3). Only an aspect ratio of 20 slightly increased the alveolar mass retained. The sensitively percent to the aspect ratio parameter was low.

**Figure 2:**
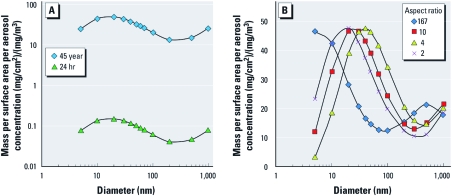
MPPD model results of alveolar mass retained per alveolar surface area per inputted aerosol concentration versus particle diameter in human lungs for (*A*) TiO_2_ and Ag NPs with exposure durations of 45 years (full working lifetime) and 24 hr, and (*B*) CNTs with aspect ratios of 167, 10, 4, and 2 after 45 years of exposure. Both *A* and *B* are based on a light exercise breathing pattern. The curves are to guide the eye.

*Concentrations recommended for* in vitro *testing.* In Figure 2, results of the deposition modeling are presented as a function of material characteristics for the two exposure scenarios of interest. Because MPPD alveolar mass retention was linearly proportional to the inputted aerosol concentration, we plotted mass per lung surface area per inputted aerosol concentration versus particle diameter. The alveolar retention per surface area for Ag and TiO_2_ spherical NPs for a full working lifetime was highest for 20-nm diameter particles (48.9 μg/cm^2^), based on an exposure aerosol concentration of 1 mg/m^3^ (Figure 2A). Relative to this peak lung surface concentration, the amount decreased to 20.3 μg/cm^2^ as size was increased to 100 nm, and also decreased to 25.1 μg/cm^2^ for 5-nm particles. For the 12-, 96- and 384-well plates used by the different assay contractors, the peak lung surface concentration equates to 186 [i.e., 48.9 μg/cm^2^ × (3.8 cm^2^/mL)], 78.2, and 54.8 μg/mL, respectively. These amounts for a full working lifetime lie within the range of the highest *in vitro* assay concentrations tested in the literature for Ag NPs and TiO_2_ NPs on human, rat, and mouse cell lines. The highest amount tested for Ag NPs ranges from 1.6 to 500 μg/mL, whereas for TiO_2_ NPs, the high-side range is 100–1,000 μg/mL or 20–520 μg/cm^2^ [see Supplemental Material, Tables S1and S2 (http://dx.doi.org/10.1289/ehp.1103750)]. Concentrations for most of the Ag NPs tested fell within 50–400 μg/mL, whereas those for TiO_2_ NPs fell within 100–250 μg/mL. Because the MPPD model uses a low estimate of alveolar surface area, a more realistic estimate would result in lower alveolar mass retained per surface area (by approximately one order of magnitude), which would correspond to a lower *in vitro* concentration for a given exposure duration. For a full working lifetime exposure duration to 0.1 mg/m^3^, the peak lung surface concentration was 4.9 μg/cm^2^ (for particles with a diameter of 20 nm) and the range was 2.0–4.9 μg/cm^2^ for particle with diameters of 5–100 nm (Figure 2A). Because alveolar retention is directly proportional to aerosol concentration, reducing the input aerosol concentration by a factor of 10 results in a linear reduction of the calculated well-plate concentration (micrograms per milliliter) by a factor of 10. The calculated well-plate concentration for a full working lifetime is similar to the low range (1.6–10.8 μg/mL) of the highest concentrations tested in *in vitro* assays for Ag NPs, but it is below the range tested for TiO_2_ NPs [see Supplemental Material, Tables S1 and S2 (http://dx.doi.org/10.1289/ehp.1103750)].

The lung surface concentration for a 24-hr exposure duration to 1 mg/m^3^ aerosol concentration of TiO_2_ or Ag NPs ranged from 0.061 to 0.15 μg/cm^2^ for particles 5–100 nm in diameter (Figure 2A). This range is more than two orders of magnitude lower than the range for a full working lifetime (Figure 2A). The peak lung surface concentration equates to 0.570, 0.240, and 0.168 μg/mL for the 12-, 96- and 384-well plates, respectively. In previous studies, the lowest amount tested for Ag NPs ranged from 0.108 to 25 μg/mL [see Supplemental Material, Table S1 (http://dx.doi.org/10.1289/ehp.1103750)], whereas for TiO_2_ NPs the range was 0.002–10 μg/mL or 0.0052–5 μg/cm^2^ (see Supplemental Material, Table S2). For 24-hr exposure duration, the alveolar surface concentrations calculated using the MPPD model fell within the range (closer to the lower end) of the lowest *in vitro* concentrations tested. Thus, the concentrations reported in previous studies (see Supplemental Material, Tables S1 and S2) are similar to the lower-bound assay test concentrations we derived using the estimated lung retention after 24 hr exposure. Each of the studies had a set exposure duration ranging from 1 to 144 hr for Ag NPs and from 5 min to 120 hr for TiO_2_ NPs (see Supplemental Material, Tables S1 and S2). Rerunning all the baseline settings for 20-nm particles for 24-hr exposure duration would require a very high aerosol concentration of approximately 330 mg/m^3^ to result in a similar peak alveolar surface concentration (~ 48.9 μg/cm^2^) for TiO_2_ and Ag NPs.

Results of the present study show that the alveolar mass retention per surface area for CNTs (with a length-to-diameter aspect ratio of 167) for a full working lifetime exposure to 1 mg/m^3^ aerosol concentration ranged from 12.4 to 46.5 μg/cm^2^ (Figure 2B), similar to the range for spherical particles. As CNT diameter decreased from 100 nm, the mass retained per surface area increased to a maximum of 46.5 μg/cm^2^ for 5-nm diameter nanotubes. In previous studies, the highest amount tested *in vitro* for CNTs ranged from 50 to 1,000 μg/mL [see Supplemental Material, Table S3 (http://dx.doi.org/10.1289/ehp.1103750)]. Most of the CNT concentrations tested fell within 50–400 μg/mL. For the more realistic aspect ratios of 4 and 10, we observed peak mass per surface area concentration at 40 nm and approximately 25 nm, respectively (Figure 2B). This peak concentration decreased with increasing diameter (Figure 2B). It is possible that the CNTs will form aggregates of larger diameter and lower aspect ratios, as reported by Baron et al. (2008). Using the model, we observed that particles with aspect ratios of approximately 20 had a maximum deposition fraction in the alveolar region. For the aspect ratios 2, 4, and 10, the mass per surface area retained for diameters > 40 nm followed a similar trend. The lung surface concentration of aspect ratio 2 was similar to the trend for spherical particles (Figure 2A) at the same aerosol concentration and exposure duration.

*Applications of approach.* The approach we took in this study was a simple screening-level assessment using the latest quantitative NM aerosol data in occupational settings to determine concentrations that may deposit and be retained deep in the human respiratory tract. The methodology we used and the alveolar retention results obtained can be generally applied to inform *in vitro* study designs, which include other NM types. Wherever possible, conservative MPPD input parameters were selected so that results would indicate a higher alveolar retention, although we attempted to choose realistic inputs as well. Our results indicate that a full lifetime occupational exposure to a concentration of 1 mg/m^3^ (one order of magnitude higher than what has typically been reported) (Table 1) is required to reach the highest concentrations currently being tested *in vitro* in most studies [see Supplemental Material, Tables S1–S3 (http://dx.doi.org/10.1289/ehp.1103750)]. Because i*n vitro* studies use different cell culture containers, in order to convert the lung surface concentrations provided here, the specific well-bottom surface area and medium volume presented to each well are required.

Note that we are comparing lung surface concentrations to concentrations being tested in a range of cell types and would expect only a small percentage of particles to reach cells in other organs of the body following absorption into the bloodstream. Nevertheless, retention of NPs in the deep lung alveolar region is important because these particles potentially can be absorbed quickly into the bloodstream. Such a phase of rapid absorption is observed immediately after inhalation, even with relatively insoluble materials (ICRP 1994). Recently, in a study in rats, Choi et al. (2010) found *a*) that NPs with hydrodynamic diameters < 6 nm and zwitterionic surface charge can rapidly enter the bloodstream from the lung and then be subsequently cleared by the kidneys; and *b*) that NPs < 34 nm with a noncationic surface charge translocate rapidly from the lung to the mediastinal lymph nodes. However, for technetium-radiolabeled 100-nm, 35-nm, and 4–20-nm diameter carbon particles, no significant systemic translocation of particles has been observed in humans (Mills et al. 2006; Möller et al. 2008; Wiebert et al. 2006). Gold NPs (5–8 nm) have been found at a low fraction (0.03–0.06% of lung concentration) in the blood of rats 1–7 days after inhalation (Takenaka et al. 2006). The type and amount of surface charge or coating may be a key factor for translocation of particles and should be evaluated. There is also a potential for larger mass amounts of NMs per lung surface area to be deposited in the tracheobronchial region. All airway surfaces may not receive the same amount of deposited particles, and localized hot spots for deposition in the vicinity of airway bifurcations have been predicted (up to 100–1,000 times higher than the average mass per surface area for particles > 10 nm) using mathematical modeling techniques (Farkas et al. 2006; U.S. EPA. 2009). However, we did not consider mass retained in this region because a large portion of the particles deposited is assumed to be cleared within 24–48 hr by action of the mucociliary escalator (U.S. EPA 2009). Potential future work will consider GI tract exposure to NPs cleared from the tracheobronchial region because it may be significant for aggregated NPs at heavy-exercise breathing conditions.

*Limitations of approach.* There are several limitations in estimating concentrations for *in vitro* testing using the latest available NP aerosol-level data from occupational settings. The instrumentation technology to measure spherical NPs typically provides nonspecific particle counts over a broad size range. For example, the SMPS provides particle counts in a size range of 2.5–1,000 nm, whereas the CPC used in several studies (Table 1) measures particles 10–1,000 nm. Particle counts become increasingly insensitive to particle sizes that are nearer to the lower limit of detection (Maynard and Aitken 2007). Measurements for nonspherical particles such as CNTs may not be reliable and may need to be corrected because these instruments are designed to count spherical particles. Additionally, to compare particle number concentration for the same type of materials across different occupational settings or manufacturing processes, the FMPS and SMPS data reported need to be normalized by dividing by the number of channels. Instrument data are not always normalized and thus may be reported as a higher count over a particle size distribution than what actually occurs. It is not currently possible to distinguish between NPs, aggregates of the same compounds, and aggregates of a mixture of particles, dust, and other airborne particle types. NPs often can agglomerate in air, which is why we present results for potentially more realistic sizes > 100 nm (Figure 2A,B). Instruments such as the universal NP analyzer (UNPA), which uses a CPC, a differential mobility analyzer (DMA), and an NP surface area monitor (NSAM) are being developed to determine the primary particle size and measure the number, surface area, and volume distributions of gas-borne NP agglomerates (Wang et al. 2010). To distinguish NPs from background particles, both real-time instrumentation measurements and qualitative analysis by electron microscopy are required (Ono-Ogasawara et al. 2009). In addition, chemical analysis is necessary for quantitatively assessing exposure to NMs at facilities with high levels of background NPs. Models are being developed to predict the change in NP number concentration for a defined source and a defined environment based on a given background aerosol concentration (Seipenbusch et al. 2008). NPs do not reach the receptor in their original size as an aerosol, but change their size and number concentration by coagulation either within the same type of materials or by interaction with a background aerosol (Seipenbusch et al. 2008).

Here we provide MPPD results in which we assumed no changes to the original aerosol concentration and performed simulations using the reported size of the particles. If particles have a tendency to aggregate and agglomerate above an aggregate size of 100 nm, the amount deposited and retained in different regions of the lung will be less (Figure 2A,B). In the case of SWCNTs, large aggregates > 10 μm in diameter can form by diffusion and van der Waals interactions between nanotubes in air or in aqueous solutions (Mutlu et al. 2010). Other drawbacks based on the method used include limitations with the MPPD model, such as a low estimate of alveolar surface area. Currently, distinctions between NM types cannot be made based on NM physicochemical characteristics. The only input possible in the latest version of the model is length-to-diameter aspect ratio for cylindrical particles. The clearance calculations in the model are based on experimental data for spherical particles, and fibers with elongated structures may have different clearance kinetics. NMs have unique physicochemical characteristics that may affect their deposition, retention, and toxicity. These characteristics include particle shape and shape distribution, large surface area:volume ratio, chemical composition and crystalline form, surface composition and coating, and surface charge. There is a need to understand which physicochemical characteristics most affect the deposition and alveolar retention of NPs and to further incorporate these key parameters into the model.

Another limitation in the approach may be the conversion of lung surface concentrations to *in vitro* test concentrations, assuming that the NMs will quickly (relative to the duration of the assay) settle onto the cells at the bottom of the well plate. If the particle transport (diffusion, sedimentation) time is slower than the *in vitro* assay testing time (which could possibly be the case for particle agglomerates, depending on their mass, size, and density) (Hinderliter et al. 2010), then the localized NM concentrations near the cells at the bottom of the well may be lower than we estimated. A recently developed computational model of particokinetics (sedimentation, diffusion) and target cell dosimetry for *in vitro* systems addressed this issue (Hinderliter et al. 2010) and could be used to calculate dose rates and target cell doses to compare with the total assay exposure time. Further, bioactivity profiles attained for NMs would need to take into account the localized concentration.

## Conclusions

Consideration of potential exposures during design of *in vitro* toxicity tests would improve interpretation of hazard screening results for use in risk assessment. The methodology described here is a first step toward improving selection of NM concentrations to test *in vitro* based on real-world inhalation exposure potential. The results obtained can be generally applied to other *in vitro* study designs and for other NM types. The approach here reveals that current high-range *in vitro* testing concentrations being used are similar to predicted lung surface area concentrations based on inhalation exposure to NMs of a high aerosol concentration in an occupational setting over the course of a full working lifetime. This methodology can be improved by better measurements of NMs in occupational settings, addition of particle property input parameters to the MPPD model, and considerations of delivered dose to cells.

## Supplemental Material

(76 KB) PDF

## References

[r1] Anjilvel S, Asgharian B. (1995). A multiple-path model of particle deposition in the rat lung.. Fundam Appl Toxicol.

[r2] Asgharian B, Hofman W, Bergmann R. (2001). Particle deposition in a multiple-path model of the human lung.. Aerosol Sci Technol.

[r3] Asgharian B, Price OT (2007). Deposition of ultrafine (NANO) particles in the human lung.. Inhal Toxicol.

[r4] Baron PA, Deye GJ, Chen BT, Schwegler-Berry DE, Shvedova AA, Castranova V (2008). Aerosolization of single-walled carbon nanotubes for an inhalation study.. Inhal Toxicol.

[r5] Bello D, Hart AJ, Ahn K, Hallock M, Yamamoto N, Garcia EJ (2008). Particle exposure levels during CVD growth and subsequent handling of vertically-aligned carbon nanotube films.. Carbon.

[r6] Bello D, Wardle BL, Yamamoto N, deVilloria RG, Garcia EJ, Hart AJ (2009). Exposure to nanoscale particles and fibers during machining of hybrid advanced composites containing carbon nanotubes.. J Nanopart Res.

[r7] Bergamaschi E. (2009). Occupational exposure to nanomaterials: present knowledge and future development.. Nanotoxicology.

[r8] Brown JS, Wilson WE, Grant LD (2005). Dosimetric comparisons of particle deposition and retention in rats and humans.. Inhal Toxicol.

[r9] Choi HS, Ashitate Y, Lee JH, Kim SH, Matsui A, Insin N (2010). Rapid translocation of nanoparticles from the lung airspaces to the body.. Nat Biotechnol.

[r10] Cohen Hubal EA (2009). Biologically relevant exposure science for 21st century toxicity testing.. Toxicol Sci.

[r11] Cohen Hubal EA, Richard A, Aylward L, Edwards S, Gallagher J, Goldsmith MR (2010). Advancing exposure characterization for chemical evaluation and risk assessment.. J Toxicol Environ Health B.

[r12] Dix DJ, Houck KA, Martin MT, Richard AM, Setzer RW, Kavlock RJ (2007). The ToxCast program for prioritizing toxicity testing of environmental chemicals.. Toxicol Sci.

[r13] Farkas A, Balashazy I, Szocs K. (2006). Characterization of regional and local deposition of inhaled aerosol drugs in the respiratory system by computational fluid and particle dynamics methods.. J Aerosol Med.

[r14] Geiser M Kreyling WG 2010 Deposition and biokinetics of inhaled nanoparticles. Part Fibre Toxicol 7 2; doi:10.1186/1743-8977-7-2 [Online 20 January 2010] 20205860 PMC2826283

[r15] Hameri K, Lahde T, Hussein T, Koivisto J, Savolainen K. (2009). Facing the key workplace challenge: assessing and preventing exposure to nanoparticles at source.. Inhal Toxicol.

[r16] Hinderliter PM Minard KR Orr G Chrisler WB Thrall BD Pounds JG 2010 ISDD: a computational model of particle sedimentation, diffusion and target cell dosimetry for *in vitro* toxicity studies. Part Fibre Toxicol 7 1 36; doi:10.1186/1743-8977-7-36 [Online 30 November 2010] 21118529 PMC3012653

[r17] Hoet PH Bruske-Hohlfeld I Salata OV 2004 Nanoparticles – known and unknown health risks. J Nanobiotechnology 2 1 12; doi:10.1186/1477-3155-2-12 [Online 8 December 2004] 15588280 PMC544578

[r18] ICRP (International Commission on Radiological Protection) (1994). Human respiratory tract model for radiological protection: a report of a task group of the International Commission on Radiological Protection. Publ no. 66.. Ann ICRP.

[r19] Judson RS, Houck KA, Kavlock RJ, Knudsen TB, Martin MT, Mortensen HM (2010). *In vitro* screening of environmental chemicals for targeted testing prioritization: the ToxCast project.. Environ Health Perspect.

[r20] Judson R, Richard A, Dix DJ, Houck K, Martin M, Kavlock R (2009). The toxicity data landscape for environmental chemicals.. Environ Health Perspect.

[r21] Kessler R. (2011). Engineered nanoparticles in consumer products: understanding a new ingredient.. Environ Health Perspect.

[r22] Klaine SJ, Alvarez PJ, Batley GE, Fernandes TF, Handy RD, Lyon DY (2008). Nanomaterials in the environment: behavior, fate, bioavailability, and effects.. Environ Toxicol Chem.

[r23] Lee JH, Lee SB, Bae GN, Jeon KS, Yoon JU, Ji JH (2010). Exposure assessment of carbon nanotube manufacturing workplaces.. Inhal Toxicol.

[r24] Maynard AD, Aitken RJ (2007). Assessing exposure to airborne nanomaterials: current abilities and future requirements.. Nanotoxicology.

[r25] Maynard AD, Baron PA, Foley M, Shvedova AA, Kisin ER, Castranova V (2004). Exposure to carbon nanotube material: aerosol release during the handling of unrefined single-walled carbon nanotube material.. J Toxicol Environ Health A.

[r26] Methner M, Hodson L, Dames A, Geraci C. (2010b). Nanoparticle emission assessment technique (NEAT) for the identification and measurement of potential inhalation exposure to engineered nanomaterials–part B: results from 12 field studies.. J Occup Environ Hyg.

[r27] Methner M, Hodson L, Geraci C. (2010a). Nanoparticle emission assessment technique (NEAT) for the identification and measurement of potential inhalation exposure to engineered nanomaterials–part A.. J Occup Environ Hyg.

[r28] Miller A, Drake PL, Hintz P, Habjan M (2010). Characterizing exposures to airborne metals and nanoparticle emissions in a refinery.. Ann Occup Hyg.

[r29] Mills NL, Amin N, Robinson SD, Anand A, Davies J, Patel D (2006). Do inhaled carbon nanoparticles translocate directly into the circulation in humans?. Am J Respir Crit Care Med.

[r30] Möller W, Felten K, Sommerer K, Scheuch G, Meyer G, Meyer P (2008). Deposition, retention, and translocation of ultrafine particles from the central airways and lung periphery.. Am J Respir Crit Care Med.

[r31] Morrow PE (1988). Possible mechanisms to explain dust overloading of the lungs.. Fundam Appl Toxicol.

[r32] Mutlu GM, Budinger GR, Green AA, Urich D, Soberanes S, Chiarella SE (2010). Biocompatible nanoscale dispersion of single-walled carbon nanotubes minimizes in vivo pulmonary toxicity.. Nano Lett.

[r33] Nanostructured & Amorphous Materials Inc (2010). Nanostructured & Amorphous Materials Inc. Homepage.. http://www.nanoamor.com/.

[r34] National Nanotechnology Initiative (2008). Strategy for Nanotechnology-Related Environmental, Health, and Safety Research. Washington, DC:Executive Office of the President, Office of Science and Technology Policy, National Nanotechnology Initiative.. http://www.nano.gov/NNI_EHS_Research_Strategy.pdf.

[r35] National Research Council (2007). Toxicity Testing in the 21st Century: A Vision and a Strategy.

[r36] NIOSH (National Institute for Occupational Safety and Health) (2005). NIOSH Current Intelligence Bulletin: Evaluation of Health Hazard and Recommendations for Occupational Exposure to Titanium Dioxide. Cincinnati, OH:NIOSH.. http://www.cdc.gov/niosh/review/public/tio2/pdfs/tio2draft.pdf.

[r37] NIOSH (National Institute for Occupational Safety and Health) (2008).

[r38] NIOSH (National Institute for Occupational Safety and Health) (2010). NIOSH Current Intelligence Bulletin: Occupational Exposure to Carbon Nanotubes and Nanofibers. Public Review Draft. Cincinnati, OH:NIOSH.. http://www.cdc.gov/niosh/docket/review/docket161A/pdfs/carbonNanotubeCIB_PublicReviewOfDraft.pdf.

[r39] Oberdörster G, Oberdörster E, Oberdörster J. (2005). Nanotoxicology: an emerging discipline evolving from studies of ultrafine particles.. Environ Health Perspect.

[r40] Ono-Ogasawara M, Serita F, Takaya M. (2009). Distinguishing nanomaterial particles from background airborne particulate matter for quantitative exposure assessment.. J Nanopart Res.

[r41] Park J, Kwak BK, Bae E, Lee J, Kim Y, Choi K (2009). Characterization of exposure to silver nanoparticles in a manufacturing facility.. J Nanopart Res.

[r42] Pauluhn J. (2010). Multi-walled carbon nanotubes (Baytubes): approach for derivation of occupational exposure limit.. Regul Toxicol Pharmacol.

[r43] Schulte PA, Murashov V, Zumwalde R, Kuempel ED, Geraci CL (2010). Occupational exposure limits for nanomaterials: state of the art.. J Nanopart Res.

[r44] Seipenbusch M, Binder A, Kasper G. (2008). Temporal evolution of nanoparticle aerosols in workplace exposure.. Ann Occup Hyg.

[r45] Sigma-Aldrich (2010). Sigma-Aldrich Homepage.. http://www.sigmaaldrich.com.

[r46] Takenaka S, Karg E, Kreyling WG, Lentner B, Möller W, Behnke-Semmler M (2006). Distribution pattern of inhaled ultrafine gold particles in the rat lung.. Inhal Toxicol.

[r47] Tsai SJ, Ada E, Isaacs JA, Ellenbecker MJ (2009). Airborne nanoparticle exposures associated with the manual handling of nanoalumina and nanosilver in fume hoods.. J Nanopart Res.

[r48] Toxic Substances Control Act of 1976 (1976).

[r49] U.S. EPA (U.S. Environmental Protection Agency) (2004). What Is the TSCA Chemical Substance Inventory? Washington, DC:U.S. EPA.. http://www.epa.gov/oppt/newchems/pubs/invntory.htm.

[r50] U.S. EPA (U.S. Environmental Protection Agency) (2009). Integrated Science Assessment for Particulate Matter (Final Report). EPA/600/R-08/139F.

[r51] U.S. EPA (U.S. Environmental Protection Agency) (2010). Nanomaterial Case Studies: Nanoscale Titanium Dioxide in Water Treatment and in Topical Sunscreen (Final). EPA/600/R-09/057F.

[r52] Wang J, Shin WG, Mertler M, Sachweh B, Fissan H, Pui DYH (2010). Measurement of nanoparticle agglomerates by combined measurement of electrical mobility and unipolar charging properties.. Aerosol Sci Technol.

[r53] Warheit DB, Sayes CM, Reed KL (2009). Nanoscale and fine zinc oxide particles: can in vitro assays accurately forecast lung hazards following inhalation exposures?. Environ Sci Technol.

[r54] Wiebert P, Sanchez-Crespo A, Falk R, Philipson K, Lundin A, Larsson S (2006). No significant translocation of inhaled 35-nm carbon particles to the circulation in humans.. Inhal Toxicol.

[r55] Yeh HC, Schum GM (1980). Models of human lung airways and their application to inhaled particle deposition.. Bull Math Biol.

