# Supplementary material for: Informing Selection of Nanomaterial Concentrations for ToxCast in Vitro Testing Based on Occupational Exposure Potential
Source: Environ Health Perspect. 2011 Jul 25;119(11):1539–46. doi: 10.1289/ehp.1103750 (PMC3226507; doi:10.1289/ehp.1103750)
Supplement: (76 KB) PDF [file ehp.1103750.s001.pdf]

SUPPLEMENTAL MATERIAL

Title: Informing Selection of Nanomaterial Concentrations for ToxCast *In Vitro* Testing based on Occupational Exposure Potential

Authors: Sumit Gangwal[1*], James S. Brown[2], Amy Wang[1], Keith A. Houck[1], David J. Dix[1], Robert J. Kavlock[1], Elaine A. Cohen Hubal[1]

Author Affiliations:

[1]National Center for Computational Toxicology (NCCT)
Office of Research and Development
U.S. Environmental Protection Agency
Research Triangle Park, NC, USA;

[2]National Center for Environmental Assessment (NCEA)
Office of Research and Development
U.S. Environmental Protection Agency
Research Triangle Park, NC, USA;

[*]CONTACT INFORMATION FOR CORRESPONDING AUTHOR:

Name: Sumit Gangwal
Email: gangwal.sumit@epa.gov
Phone: 919-541-3864
Fax: 919-541-1194
Address: Mail Drop D-343-03, US EPA, Research Triangle Park, NC, 27711

Table of Contents

Supplemental Material, Table S1 ……….……..…………………………………………... Page 2

Supplemental Material, Table S2 ……..……..………………………………………….. Page 3

Supplemental Material, Table S3 ………..………...…………………………………..….. Page 4

References ………………….....…………………..……………………………………… Page 5

Supplemental Material, Table S1. Examples of silver (Ag) nanoparticle *in vitro* testing concentrations.

**Cells**	**Reported testing concentrations**	**Exposure duration**	**NM size and coating information**	**References**
Mouse primary fibroblasts and primary liver cells	1.56, 3.12, 6.25, 12.5, 25, 50, 100, 200, 300, 400, 500 µg/mL	24 h	16.6 nm average, with > 90% in 7–20 nm range. No coating info	(Arora et al. 2009)
Mouse peritoneal macrophage cell line (RAW264.7)	0.2, 0.4, 0.8, 1.6 ppm (or µg/mL)	24, 48, 72, 96 h	68.9 nm average in culture media; No coating info	(Park et al. 2010)
Mouse embryonic stem (mES) cells and mouse embryonic fibroblasts (MEF)	50 µg/mL	4, 24, 48, 72 h	25 nm for both uncoated and Polysaccharide-coated Ag	(Ahamed et al. 2008)
Rat liver derived cell line (BRL 3A)	2.5, 5, 10, 25, 50 µg/mL	2, 24 h	15, 100 nm; No coating info	(Hussain et al. 2005)
Human lung fibroblast cell line (IMR-90) and human glioblastoma cells line (U251)	25, 100, 200, 400 µg/mL	2, 6, 24, 48 h	6-20 nm; Soluble potato starch stabilized	(AshaRani et al. 2009)
Rat neuronal cell line derived from adrenal medulla (PC12)	0.108, 0.324, 1.08, 3.24, 10.8 µg/mL (equates to 1, 3, 10, 30, 100 µM nominal Ag—concentration if all Ag were freely dissolved)	1, 24, 96, 144 h	6 nm average (with 85% < 10 nm) for Citrate-coated Ag; 21 nm (with 88% < 25 nm) and 75 nm (with 57% < 81 nm) for Polyvinylpyrrolidone (PVP)-coated Ag	(Powers et al. 2011)

Supplemental Material, Table S2. Examples of titanium dioxide (TiO_2) nanoparticle *in vitro* testing concentrations.

**Cells**	**Reported testing concentrations**	**Exposure duration**	**NM size and form information**	**References**
Human Bronchial Epithelial cell line, (16HBE14o-)	5, 10, 20 µg/cm^2	4, 24 hr	12 nm average by TEM, 86 and 356 nm average hydrodynamic diameter of resolved particle in culture medium (99.9% anatase); 48 nm average by TEM, 243 nm average hydrodynamic diameter of resolved particle in culture medium (65% anatase, 35% rutile)	(Hussain et al. 2009)
Rat liver cell line (BRL 3A)	10, 50, 100, 250 µg/mL	24 h	40 nm; No form info	(Hussain et al. 2005)
Human alveolar epithelial cell line (A5459)	5, 10, 50, 100, 200 µg/mL	1, 24 h	30 nm mean diameter; No form info	(Park et al. 2007)
Primary human neutrophils	0.002, 0.02, 0.2, 2 10, 20, 50, 100 µg/mL	5 min , 15 min, 1 h, 24 h	No size info; Anatase	(Goncalves et al. 2010)
Primary fibroblasts from *gpt* delta transgenic mouse embryo	0.1, 1, 10, 30, 60, 100 µg/mL	24, 72 h	5 nm or 40 nm average primary particle diameter; Both are anatase	(Xu et al. 2009)
Mouse macrophage cell line (RAW264.7)	0.0052, 0.052, 0.52, 5.2, 52, 520 µg/cm^2	24 h	30 nm; Rutile form	(Kim et al. 2009)
Mouse testis Leydig cell line (TM3)	1, 10, 30, 100, 1000 µg/mL	24, 48, 72, 96, 120 h	25–70 nm; No form info	(Komatsu et al. 2008)

Supplemental Material, Table S3. Examples of carbon nanotube *in vitro* testing concentrations.

**Cells**	**Reported testing concentrations**	**Exposure duration**	**NM size and functional group information**	**References**
Primary neonatal rat ventricular cardiomyocytes	0.25, 2.5, 25, 50 μg/mL	24 h	SWCNT; No size or functional group info	(Helfenstein et al. 2008)
Primary human epidermal keratinocytes, cryopreserved	0.1, 0.2, 0.4 mg/mL	1, 2, 4, 8, 12, 24, 48 h	MWCNT, 100 nm average diameter, 3.6-50 μm length; No functional group info	(Monteiro-Riviere et al. 2005)
Human astrocyte (from astocytoma) (D384) and human lung alveolar type II cells (from adenocaricnoma) (A549)	1, 10, 100, 200, 400, 800 μg/mL	24, 48 h	All MWCNTs with 20-30 nm outer diameter, 1-2 nm wall thickness; MWCNT, 500–2000 nm length; MWCNT-COOH, 100–300 nm length; MWCNT-NH_2, 100–300 nm length; hf-MW-NH_2, 50–100 nm length	(Coccini et al. 2010)
Mouse macrophages (RAW 264.7) and murine bone marrow-derived dendritic cells (bmDC)	3, 10, 30, 300 μg/mL	2, 6, 24, 48 h	SWCNT, < 2 nm diameter, 1–5 μm length, No functional group info; MWCNT, 10–30 nm diameter, 1–2 μm length, No functional group info	(Palomäki et al. 2010)
3T3 fibroblasts , macrophages (RAW 264.7), telomerase-immortalized human bronchiolar epithelial cells (hT)	10, 100, 1000 μg/mL	2, 3, 4, 6, 10, 12, 18, 20, 24 h	All MWCNT with 0.5-2 μm length, Three different diameter ranges: 1) inner diameter 2-5 nm, outer diameter < 8 nm, 2) inner diameter 5-10 nm, outer diameter 20-30 nm, 3) inner diameter 5-15 nm, outer diameter > 50 nm; No functional group info	(Sohaebuddin et al. 2010)

References

Ahamed M, Karns M, Goodson M, Rowe J, Hussain SM, Schlager JJ, et al. 2008. DNA damage response to different surface chemistry of silver nanoparticles in mammalian cells. Toxicol Appl Pharm 233(3):404-410.

Arora S, Jain J, Rajwade JM, Paknikar KM. 2009. Interactions of silver nanoparticles with primary mouse fibroblasts and liver cells. Toxicol Appl Pharm 236(3):310-318.

Asharani PV, Hande MP, Valiyaveettil S. 2009. Anti-proliferative activity of silver nanoparticles. BMC Cell Biol 10: 65.

Coccini T, Roda E, Sarigiannis DA, Mustarelli P, Quartarone E, Profumo A, et al. 2010. Effects of water-soluble functionalized multi-walled carbon nanotubes examined by different cytotoxicity methods in human astrocyte D384 and lung A549 cells. Toxicology 269(1):41-53.

Goncalves DM, Chiasson S, Girard D. 2010. Activation of human neutrophils by titanium dioxide (TiO_2) nanoparticles. Toxicol In Vitro 24(3):1002-1008.

Helfenstein M, Miragoli M, Rohr S, Muller L, Wick P, Mohr M, et al. 2008. Effects of combustion-derived ultrafine particles and manufactured nanoparticles on heart cells in vitro. Toxicology 253(1-3):70-78.

Hussain S, Boland S, Baeza-Squiban A, Hamel R, Thomassen LC, Martens JA, et al. 2009. Oxidative stress and proinflammatory effects of carbon black and titanium dioxide nanoparticles: Role of particle surface area and internalized amount. Toxicology 260(1-3):142-149.

Hussain SM, Hess KL, Gearhart JM, Geiss KT, Schlager JJ. 2005. In vitro toxicity of nanoparticles in BRL 3A rat liver cells. Toxicol In Vitro 19(7):975-983.

Kim HW, Ahn EK, Jee BK, Yoon HK, Lee KH, Lim Y. 2009. Nanoparticulate-induced toxicity and related mechanism in vitro and in vivo. J Nanopart Res 11(1):55-65.

Komatsu T, Tabata M, Kubo-Irie M, Shimizu T, Suzuki K, Nihei Y, et al. 2008. The effects of nanoparticles on mouse testis Leydig cells in vitro. Toxicology In Vitro 22(8):1825-1831.

Monteiro-Riviere NA, Nemanich RJ, Inman AO, Wang YY, Riviere JE. 2005. Multi-walled carbon nanotube interactions with human epidermal keratinocytes. Toxicol Lett 155(3):377-384.

Palomäki J, Karisola P, Pylkkänen L, Savolainen K, Alenius H. 2010. Engineered nanomaterials cause cytotoxicity and activation on mouse antigen presenting cells. Toxicology 267(1-3):125-131.

Park EJ, Yi J, Kim Y, Choi K, Park K. 2010. Silver nanoparticles induce cytotoxicity by a Trojan-horse type mechanism. Toxicology In Vitro 24(3):872-878.

Park S, Lee YK, Jung M, Kim KH, Chung N, Ahn EK, et al. 2007. Cellular toxicity of various inhalable metal nanoparticles on human alveolar epithelial cells. Inhal Toxicol 19 Suppl 1:59-65.

Powers CM, Badireddy AR, Ryde IT, Seidler FJ, Slotkin TA. 2011. Silver nanoparticles compromise neurodevelopment in PC12 cells: critical contributions of silver ion, particle size, coating, and composition. Environ Health Perspect 119(1):37-44.

Sohaebuddin SK, Thevenot P, Baker D, Eaton JW, Tang L. 2010. Nanomaterial cytotoxicity is composition, size, and cell type dependent. Part Fibre Toxicol 7(1):22.

Xu A, Chai Y, Hei TK. 2009. Genotoxic responses to titanium dioxide nanoparticles and fullerene in *gpt* delta transgenic MEF cells. Part Fibre Toxicol 6:3.
